# Microbial dark matter coming to light: challenges and opportunities

**DOI:** 10.1093/nsr/nwaa280

**Published:** 2020-12-08

**Authors:** Jian-Yu Jiao, Lan Liu, Zheng-Shuang Hua, Bao-Zhu Fang, En-Min Zhou, Nimaichand Salam, Brian P Hedlund, Wen-Jun Li

**Affiliations:** State Key Laboratory of Biocontrol, Guangdong Provincial Key Laboratory of Plant Resources and Southern Marine Science and Engineering Guangdong Laboratory (Zhuhai), School of Life Sciences, Sun Yat-Sen University, China; State Key Laboratory of Biocontrol, Guangdong Provincial Key Laboratory of Plant Resources and Southern Marine Science and Engineering Guangdong Laboratory (Zhuhai), School of Life Sciences, Sun Yat-Sen University, China; State Key Laboratory of Biocontrol, Guangdong Provincial Key Laboratory of Plant Resources and Southern Marine Science and Engineering Guangdong Laboratory (Zhuhai), School of Life Sciences, Sun Yat-Sen University, China; Department of Environmental Science and Engineering, University of Science and Technology of China, China; State Key Laboratory of Biocontrol, Guangdong Provincial Key Laboratory of Plant Resources and Southern Marine Science and Engineering Guangdong Laboratory (Zhuhai), School of Life Sciences, Sun Yat-Sen University, China; State Key Laboratory of Desert and Oasis Ecology, Xinjiang Institute of Ecology and Geography, Chinese Academy of Sciences, China; State Key Laboratory of Biocontrol, Guangdong Provincial Key Laboratory of Plant Resources and Southern Marine Science and Engineering Guangdong Laboratory (Zhuhai), School of Life Sciences, Sun Yat-Sen University, China; State Key Laboratory of Biocontrol, Guangdong Provincial Key Laboratory of Plant Resources and Southern Marine Science and Engineering Guangdong Laboratory (Zhuhai), School of Life Sciences, Sun Yat-Sen University, China; School of Life Sciences, University of Nevada Las Vegas, USA; Nevada Institute of Personalized Medicine, University of Nevada Las Vegas, USA; State Key Laboratory of Biocontrol, Guangdong Provincial Key Laboratory of Plant Resources and Southern Marine Science and Engineering Guangdong Laboratory (Zhuhai), School of Life Sciences, Sun Yat-Sen University, China; State Key Laboratory of Desert and Oasis Ecology, Xinjiang Institute of Ecology and Geography, Chinese Academy of Sciences, China

Microbes are the most abundant and diverse cellular life forms on Earth and colonize a wide range of environmental niches. However, more than 99% of bacterial and archaeal species have not been obtained in pure culture [1] and we have only glimpsed the surface of this mysterious microbial world. This is so-called Microbial Dark Matter (MDM): the enormous diversity of yet-uncultivated microbes that microbiologists can only study by using cultivation-independent techniques. Recently, a number of international projects have dramatically increased our understanding of the extent and distribution of microbial diversity, including the Global Catalogue of Microorganisms (GCM), the Genomic Encyclopedia of Bacteria and Archaea (GEBA), the Earth Microbiome Project (EMP), the Genomic Encyclopedia of Bacteria and Archaea-Microbial Dark Matter (GEBA-MDM) and several primate microbiome projects; however, the functional diversity of MDM is still mysterious. This perspective addresses why MDM deserves scientific effort and illustrates challenges and opportunities in the future study of these enigmas.

## TECHNOLOGY BRINGS LIGHT TO MDM

Our planet harbors a vast repository of diverse microorganisms. More than 20 000 species (<1%) of bacterial and archaeal species have been described and validly published [2], yet our nascent understanding of the biology of some of the major branches of the tree of life limits our understanding of the microbial world. Until recent decades, microbiology research was mostly focused on pure cultures. Only with the development of new advancements in DNA sequencing and computing has the great challenge of the genomic exploration of MDM directly from complex environmental samples been feasible. Notably, single-amplified genomes (SAGs) and metagenome-assembled genomes (MAGs) obtained from single-cell genomic and metagenomic approaches, respectively, have become the most effective methods that enable organism-level genomic analysis of complex microbial ecosystems without the need for cultivation, which brings light to MDM.

**Figure 1.**
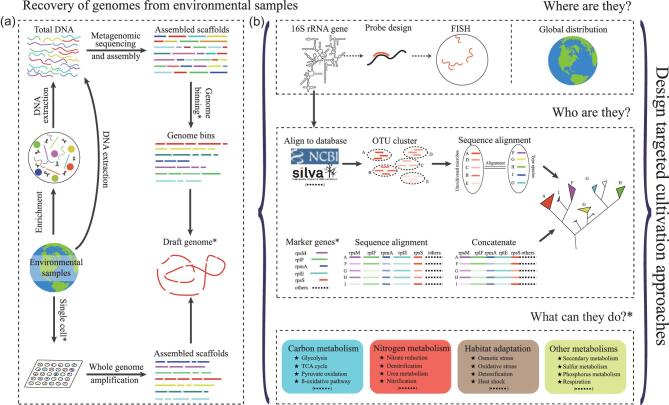
Schematic of research approaches to answer the basic scientific questions about MDM. (a) Typical pipeline to produce SAGs or MAGs. Single-cell isolation requires specialized instrumentation, such as flow cytometry, microfluidics or micromanipulators, and clean rooms are also required for downstream handling. Genome binning tools are available, such as MetaBAT, MaxBin, GroopM, MetaWatt and CONCOCT. The quality of draft genomes could follow the minimum standards information about SAGs and MAGs, which was proposed by the Genome Standards Consortium to facilitate communication and more robust comparative genomic analyses of microbial diversity [3]. (b) Downstream analysis based on MDM genomes and a flowchart illustrating how genomic data can be integrated. Marker genes used for phylogenetic analysis are flexible enough to accommodate changes over time. Combination of multi-omics, FISH, MAR, NanoSIMS, Raman and other techniques would provide compelling evidence for microbial function and the framework to design targeted cultivation strategies.

SAGs are typically accessed by sequencing amplified genomic DNA from individual cells using a variety of whole-genome amplification (WGA) technologies. MAGs, on the other hand, are recovered from metagenomic data sets using computational binning tools (Fig. 1). With high-quality SAGs or MAGs, the metabolic potential of MDM can be more reliably predicted compared to functions extrapolated from phylogenetic affiliation based on marker genes (e.g. through 16S rRNA gene phylogeny) or from medium- or low-quality draft genomes [3]. However, even low-quality MAGs and SAGs can give researchers insight into three basic scientific questions: who are they, where are they and what can they do? Armed with this information, microbiologists can readily design experiments to probe *in situ* physiology and design targeted cultivation approaches.

## MDM: A BAG FULL OF SURPRISES

Studies on SAGs and MAGs have unveiled some big surprises on the diversity of microbes in nature and facilitated major discoveries. During the last decade, a number of MAGs or SAGs representing major lineages have been retrieved from environmental samples (Table 1). A few landmark papers on systematic and evolutionary microbiology are as follows. Hug *et al.* used more than 1000 MDM genomes, together with public genomic data, to infer the tree of life and described a hyper-diverse clade of MDM termed the Candidate Phyla Radiation (CPR), subdividing the domain Bacteria [4]. Parks *et al.* recovered nearly 8000 MAGs from more than 1500 metagenomic data sets to reconstruct an expanded version of the tree of life, and increased the prokaryotic phylogenetic diversity by more than 30%, including 20 novel phyla of Bacteria and Archaea [5]. Recent research has leaned on MAG and SAG data sets to explore the Asgard archaea and propose new and exciting models on the origin of eukaryotic cellular complexity [6]. Specifically, the entangle-engulf-endogenize model for eukaryogenesis has been proposed based on the study of the decade-long enrichment and isolation of the Asgard archaeon ‘*Candidatus* Prometheoarchaeum syntrophicum’ [7].

These studies of MDM are changing the way we think about the origin and evolution of life. Another notable research area that deserves scientific effort is the metabolic versatility of MDM, which has uncovered significant surprises in recent years. For example, genes encoding enzymes for anaerobic methane oxidation and dissimilatory sulfate reduction were detected in *Korarchaeota* MAGs, suggesting these two functions, typically encoded by different partners of a syntrophy, are coupled within a single organism [8]. Additionally, genomic analysis of MDM has opened our eyes to new microbial functions that can be applied for advancements in biomedicine, bioenergy and biotechnology, and bioremediation. Functional versatility of MDM shows us its ‘colorful’ side, surprises us and gives us a better understanding of the world in which we live.

## CHALLENGES AND OPPORTUNITIES

The current ease of DNA sequencing provides ample genomic information for exploring MDM, transporting MDM science from a data-poor past to a data-rich era. The volume of genomic data in public databases will continue to increase, and more and more MDM will be discovered and probed. How to maximize the utility of big data will continue to be at the forefront of research in microbiology, and the time is right to consider what we should do in the post-genomic era. Here, we outline three major challenges of MDM research.

The first challenge is to communicate better. Communication about MDM is currently difficult because the taxonomy and nomenclature are unstable. The current iteration of the *International Code of Nomenclature of Prokaryotes (ICNP)*, which governs bacterial and archaeal nomenclature, does not cover uncultivated microorganisms, making scientific communication challenging. There is no doubt that instability and synonymy in microbial nomenclature lead to confusion in the scientific community and beyond (e.g. agriculture, law, biotechnology and medicine). Therefore, a system of rules to name, compile and communicate about MDM is urgently required. Toward this end, genome sequences have been proposed to replace or complement pure cultures to serve as type material for taxonomic descriptions of prokaryotes and an independent nomenclatural system for MDM has been recommended [9]. Recently, a roadmap for naming uncultivated Archaea and Bacteria was proposed with the goal of working toward a stable nomenclature and taxonomy for all microorganisms, including those that have not been cultivated [10]. However, to date, these recommendations still require engagement from the scientific community [10]. The next meeting of the Bergey's International Society for Microbial Systematics (BISMiS), hosted in Guangzhou (China), in November 2022 will focus on the nomenclature of uncultivated microorganisms, with a goal of generating international consensus.

**Table 1.** Candidate new phyla revealed by using MAGs or SAGs.

Candidate phylum	MAG or SAG	First description	Reference^*^
Acetothermia (OP1/KB1 group)	MAG, SAG	Obsidian Pool, Yellowstone National Park	Hugenholtz *et al.*, 1998; Rinke *et al.*, 2013
Aenigmarchaeota (DSEG)^a^	SAG	Homestake Mine	Rinke *et al.*, 2013
Aerophobetes (CD12)	SAG	Sakinaw Lake	Rinke *et al.*, 2013
Aigarchaeota (pSL4; HWCG-I)^a^	MAG, SAG	Geothermal water stream from a subsurface mine in Japan	Takami *et al.*, 2012; Rinke *et al.*, 2013
Aminicenantes (OP8)	SAG	Obsidian Pool, Yellowstone National Park	Hugenholtz *et al.*, 1998; Rinke *et al.*, 2013
Atribacteria (OP9/JS1)	MAG, SAG	Obsidian Pool, Yellowstone National Park	Hugenholtz *et al.*, 1998; Dodsworth *et al.*, 2013; Rinke *et al.*, 2013
Bathyarchaeota (MCG)^a^	MAG	Marine sediment	Lloyd *et al.*, 2013
BD1–5	MAG	Groundwater samples	Wrighton *et al.*, 2012
Berkelbacteria (ACD58)	MAG	Aquifer adjacent to the Colorado River	Wrighton *et al.*, 2014
BRC1	SAG	Etoliko Lagoon and Sakinaw Lake	Rinke *et al.*, 2013
Calescamantes (EM19)	SAG	Great Boiling Spring and Gongxiaoshe hot spring	Rinke *et al.*, 2013
Cloacimonetes (WWE1)	MAG, SAG	Municipal Anaerobic Sludge Digester	Pelletier *et al.*, 2008; Rinke *et al.*, 2013
CPR (RIF1–46 and SM2F11)	MAG	Aquifer sediments and groundwater, USA	Anantharaman *et al.*, 2016
Diapherotrites (pMC2A384)^a^	SAG	Homestake Mine	Rinke *et al.*, 2013
EM3 (former OP2)	SAG	Obsidian Pool, Yellowstone National Park	Hugenholtz *et al.*, 1998; Rinke *et al.*, 2013
Fervidibacteria (OctSpA1–106)	SAG	Octopus Spring sediment	Rinke *et al.*, 2013
Geoarchaeota^a^	MAG	Acidic iron mats in Yellowstone National Park	Kozubal *et al.*, 2013
Gracilibacteria (GN02)	MAG, SAG	Guerrero Negro hypersaline microbial mat	Wrighton *et al.*, 2012; Rinke *et al.*, 2013
Heimdallarchaeota^a^	MAG	Marine sediments (Loki's Castle and Aarhus Bay)	Zaremba-Niedzwiedzka *et al.*, 2017
Hydrogenogenetes (BRC1/NKB19)	MAG, SAG	Bulk soil and rice roots	Rinke *et al.*, 2013
Korarchaeota^a^	MAG	Obsidian Pool, Yellowstone National Park	Hugenholtz *et al.*, 1998
Kryptonia^b^	MAG	High-temperature pH-neutral geothermal springs	Eloe-Fadrosh *et al.*, 2016
KSB3	MAG	Anaerobic wastewater treatment bioreactor	Sekiguchi *et al.*, 2015
Latescibacteria (WS3)	SAG	Wurtsmith Air Force Base, Michigan	Dojka *et al.*, 1998; Rinke *et al.*, 2013;
Lokiarchaeota^a^	MAG	Arctic Mid-Ocean Ridge	Spang *et al.*, 2015
Marinimicrobia (SAR406)	MAG, SAG	Subsurface of Atlantic and Pacific oceans	Rinke *et al.*, 2013
Melainabacteria	MAG	Human gut and groundwater	Di *et al.*, 2013
Microgenomates (OP11)	MAG, SAG	Obsidian Pool, Yellowstone National Park	Hugenholtz *et al.*, 1998; Wrighton *et al.*, 2012
Nanoarchaeota^a^	MAG	Submarine hot vent	Huber *et al.*, 2002
Nanohaloarchaeota^a^	MAG, SAG	Ponds of Bras del Port salterns, Spain	Ghai *et al.*, 2011
Nezhaarchaeota^a^^,^^b^	MAG	Jinze Hot Spring, Yunnan, China	Yinzhao *et al.*, 2019
NC10	MAG	Aquatic microbial formations in flooded caves	Ettwig *et al.*, 2010
Odinarchaeota^a^	MAG	Hot spring (Yellowstone National Park and Radiata Pool)	Zaremba-Niedzwiedzka *et al.*, 2017
Omnitrophica (OP3)	MAG, SAG	Obsidian Pool, Yellowstone National Park	Hugenholtz *et al.*, 1998
Pacearchaeota^a^	MAG	Aquifer adjacent to the Colorado River	Castelle *et al.*, 2015
Parcobacteria (OD1)	MAG, SAG	Obsidian Pool, Yellowstone National Park	Hugenholtz *et al.*, 1998; Wrighton *et al.*, 2012
Parvarchaeota (ARMAN)^a^	MAG, SAG	A drift of the Richmond Mine, Northern California	Rinke *et al.*, 2013;
PER	MAG, SAG	Groundwater samples	Wrighton *et al.*, 2012
Poribacteria	SAG	Marine sponge-associated	Siegl *et al.*, 2011
Saccharibacteria (TM7)	MAG, SAG	Peat bog (TM means Torf, Mittlere schicht)	Rheims *et al.*, 1996; Marcy *et al.*, 2007
SBR1093^b^	MAG	Activated sludge from wastewater treatment system	Wang *et al.*, 2014
SR1	MAG, SAG	Hydrocarbon-contaminated aquifer (SR, ‘Sulfur River’)	Kantor *et al.*, 2013
Tectomicrobia	MAG, SAG	Marine sponge	Wilson *et al.*, 2014
Thorarchaeota^a^	MAG	White Oak River estuary sediments	Seitz *et al.*, 2016
TM6	SAG	Peat bog (TM means Torf, Mittlere schicht)	Rheims *et al.*, 1996; McLean *et al.*, 2013
UAP1–3^a^	MAG	Assembled from public metagenomes	Parks *et al.*, 2017
UBP1–17	MAG	Assembled from public metagenomes	Parks *et al.*, 2017
Verstraetearchaeota^a^	MAG	Cellulose-degrading anaerobic digesters	Vanwonterghem *et al.*, 2016
Woesearchaeota^a^	MAG	Aquifer adjacent to the Colorado River	Castelle *et al.*, 2015
WS1	SAG	Wurtsmith Air Force Base, Michigan	Dojka *et al.*, 1998; Rinke *et al.*, 2013;
WWE3	MAG	Anaerobic sludge digester	Wrighton *et al.*, 2014

^a^The archaeal candidate phyl.

^b^The phyla published with Chinese participation.

^*^Supplementary data.

The second challenge is that it remains very difficult to extrapolate functions from genomic information. Metabolic predictions based on genomic information from MAGs and SAGs of MDM is only potential rather than strong evidence, and more than half of the genes from MDM genomes cannot be assigned to any annotated functions, severely limiting our understanding of the functions of MDM. Therefore, in the post-genomic era, the study of the functions of MDM will be needed to advance this line of research. As functional research has always been strongly influenced by cultivation, growing efforts have been devoted to using microbiological and bioinformatics tools to bridge the gap between MDM and their function. Metatranscriptomics, metaproteomics and metabolomics approaches have been used to detect the expression of genes and biochemical pathways and to study the dynamics of metabolites. Coupled with these omics approaches, other tools such as fluorescence *in situ* hybridization (FISH), microautoradiography (MAR), nanometer-scale secondary-ion mass spectrometry (nanoSIMS), and even Raman techniques can reveal assimilatory functions of specific MDM species. Furthermore, the synthesis and heterologous expression of specific functional genes of MDM is also an effective method for assessing the function of one or more genes of interest.

The third challenge is that cultivation of MDM remains difficult. Cultures enable unambiguous assignment of physiological traits to the purified organisms and serve as important resources for the research community. Despite advances in cultivation-independent approaches in microbiology, the isolation of recalcitrant MDM is inherently challenging. Fortunately, insights from SAGs and MAGs, multi-omics approaches and various function-based labeling techniques such as MAR and nanoSIMs, can provide the framework necessary for testing hypotheses to link species to functions, and to rationally design targeted cultivation strategies. It is worth noting that microbial interactions are often ignored in cultivation method design, which hampers attempts to cultivate MDM with syntrophic partners. Therefore, data-supported networks, such as co-occurrence networks from 16S rRNA gene data sets, and functional networks from multi-omics data, would provide great opportunities for guiding the design of cultivation strategies. Overall, we still have unlimited possibilities for the creative application of novel approaches to cultivate and isolate novel microorganisms.

## THE FUTURE OF MDM RESEARCH

Earth is inhabited by an enormous diversity of microbes, most of which are still uncultivated, and their ecological functions are poorly known. In ‘Challenges and Opportunities’ above, we highlighted three major challenges regarding MDM: to find out who they are, where they are and what they can do. We advocate three approaches to promote future MDM research. First, large-scale investigations of MDM integrating various omics approaches (i.e. metagenomics, metatranscriptomics, metaproteomics, metabolomics and culturomics) are still needed to explore a variety of natural environments, especially extreme habitats, which will stretch our imaginations with regard to the taxonomic and functional diversity of microbial life. Second, we need to strengthen connections between species and their functions *in situ*. This can now be studied by integrating omics, FISH, MAR, NanoSIMS, Raman and heterologous expression techniques, which offer insights into mechanisms of species. Third, we need to establish and explore the new cultivation approaches guided by multi-omics information and network approaches, in order to test hypotheses and obtain enrichments or pure cultures. Further understanding of MDM requires a combination of many disciplines and multidimensional knowledge; we also highlight the need to establish MDM research centers to tackle these challenges in different ways, from developing research facilities and providing technical assistance, to training the next generation of scientists, and providing academic communication platforms. The exploration of MDM is likely to open a magic box, in which a bag full of surprises could be displayed. The study of MDM is still in its infancy and further exploration of MDM will continue to provide unanticipated and exciting answers about the evolution and roles of MDM in nature. However, as a scientist, the wonderful things displayed by the ‘box’ are not the final answer of MDM research but rather a starting point for further exploration of ecological and evolutionary mechanisms.
